# Ligand Conjugated Multimeric siRNAs Enable Enhanced Uptake and Multiplexed Gene Silencing

**DOI:** 10.1089/nat.2019.0782

**Published:** 2019-09-26

**Authors:** Jonathan M. Brown, James E. Dahlman, Kristin K. Neuman, Carla A.H. Prata, Monika C. Krampert, Philipp M. Hadwiger, Hans-Peter Vornlocher

**Affiliations:** ^1^ MPEG LA, Chevy Chase, Maryland.; ^2^ Wallace H. Coulter Department of Biomedical Engineering, Atlanta, Georgia.; ^3^ AxoLabs GmbH, Kulmbach, Germany.

**Keywords:** multimer, oligonucleotide, enhanced, knockdown, GalNAc, TTR

## Abstract

Small interfering RNAs (siRNAs) conjugated to *N*-acetylgalactosamine (GalNAc) ligands have been used to treat disease in patients. However, conjugates with other ligands deliver siRNA less efficiently, limiting the development of new targeted therapies. Most approaches to enhancing the potency of such conjugates have concentrated on increasing ligand effectiveness and/or the chemical stability of the siRNA drug. One complementary and unexplored alternative is to increase the number of siRNAs delivered per ligand. An ideal system would be a single chemical entity capable of delivering multiple copies of an oligonucleotide drug and/or several such drugs simultaneously. Here we report that siRNAs can be stably linked together under neutral aqueous conditions to form chemically defined siRNA “multimers,” and that these multimers can be delivered *in vivo* by a GalNAc ligand. Conjugates containing multiple copies of the same siRNA showed enhanced activity per unit of ligand, whereas siRNAs targeting different genes linked to a single ligand facilitated multigene silencing *in vivo*; this is the first demonstration of silencing several genes simultaneously *in vivo* using ligand-directed multimeric siRNA. Multimeric oligonucleotides represent a powerful and practical new approach to improve intracellular conjugate delivery.

## Introduction

A small interfering RNA (siRNA) drug delivered to hepatocytes using an ionizable lipid nanoparticle (LNP) has been recently approved by the Food and Drug Administration [1,2] demonstrating the utility of RNA therapeutics when the drugs can be efficiently delivered to target cells. More recently, clinical data have been generated using a conjugate-based siRNA drug delivery system based on *N*-acetylgalactosamine (GalNAc) [3]. GalNAc binds to the asialoglycoprotein receptor (ASGPR), which is (i) specifically expressed on hepatocytes at high levels, and (ii) rapidly endocytosed upon binding. Although the clinical data generated using GalNAc-based delivery systems are promising, target mRNA silencing by LNPs involving siRNA doses lower by an order of magnitude or more suggest that there is an opportunity to substantially improve the efficiency of GalNAc delivery [3–5]. To this end, improvements have been made to (i) the binding characteristics of the GalNAc ligands [6] by preparation in triantennary form to enhance avidity, (ii) the stability to nucleases of the siRNA [7,8] by use of chemically modified nucleotides, and the biological interactions between GalNAc and ASGPR have been studied [9] in detail. More generally, there is a need to improve the efficiency of other conjugate delivery systems, including those utilizing antibodies [10], peptides [11], synthetic amphiphiles [12], lipids [13], aptamers [14], and other ligands to target cell types other than hepatocytes. Improving the delivery of conjugate-based delivery is a fundamental challenge in the field of oligonucleotide therapies.

One unexplored alternative that would complement all these advances is to increase the number of siRNAs that are delivered per ligand. This would require the synthesis of a defined number of siRNAs linked to one another and to the ligand. We reasoned this “defined multimeric” approach would be advantageous for at least two reasons. First, it would increase the amount of siRNA delivered into the cytoplasm per ligand–target interaction. This advantage will be more important for ligands that may have less efficient binding and endocytosis characteristics than GalNAc-ASGPR. Second, it would enable multigene silencing facilitated by a single, chemically defined, ligand-directed siRNA drug, which has not been reported to date. Most diseases and phenotypes are driven by combinations of genes and while up to five siRNAs have been delivered concurrently *in vivo* [15–18], these multigene silencing experiments have been performed by formulating siRNA mixtures into LNPs. As the siRNAs are not chemically linked, the LNPs may be difficult to prepare with a uniform siRNA content and profile. By contrast, a covalently linked ligand-multimeric siRNA can be isolated as a single chemical entity.

Herein we report the synthesis and chemical characterization of such constructs, which we term “defined multimers,” consisting of a specific number of oligonucleotides (eg, two or more identical and/or differing siRNAs), that are conjugated to a targeting ligand. We also report the biological activities of a series of homo- and heteromultimers prepared using a novel intermediate and subsequent asymmetric annealing steps. Of note, this multimeric approach is not ligand or receptor specific, nor is it specific to siRNA; it can be applied to other conjugate systems, and other oligonucleotide drug types either singly or in combination.

## Materials and Methods

### siRNA monomer synthesis

Monomeric oligoribonucleotides were synthesized using standard phosphoramidite chemistry on controlled pore glass (CPG) supports. 2′-Deoxyribo-, 2′-O-methylribo-, and 2′-deoxy-2′-fluororibo-phosphoramidites were obtained from SAFC Proligo (Hamburg, Germany). 5′- and 3′-aminohexyl linkers were introduced by TFA-protected hexylamino-linker phosphoramidite (Sigma-Aldrich, SAFC, Hamburg, Germany) and phthalimido protected hexylamino-linker immobilized on CPG (Prime Synthesis, Aston, PA), respectively. 3′- or 5′-terminal thiol groups were introduced by 1-O-dimethoxytrityl-hexyl-disulfide, 1′-[(2-cyanoethyl)-(N,N-diisopropyl)]-phosphoramidite (NucleoSyn, Olivet Cedex, France). 5-Ethylthio-1H-tetrazole (ETT; 0.5 M in acetonitrile) was used as activator for all couplings. Phosphorothioate linkages were introduced using 3-[(dimethylamino-methylidene)amino]-3H-1,2,4-dithiazole-3-thione (DDTT). Disulfide containing oligonucleotides (1 mM in triethylammonium bicarbonate [TEAB] buffer, 100 mM, pH 8.5) were reduced using dithiothreitol (50–100-fold excess) and the reaction monitored by high-performance liquid chromatography (HPLC). Excess reagent and byproducts were removed using size exclusion chromatography (HiPrep column) and water as eluant. The Key Figure contains definitions of abbreviations used in (nearly) all the figures and schemes.

Monomeric oligonucleotides were isolated by standard cleavage/deprotection protocols followed by anion exchange (AEX) HPLC (20 mM Tris, 1 mM ethylenediaminetetraacetic acid [EDTA], pH 7.4 in 20% aqueous acetonitrile, sodium perchlorate gradient from 10 to 500 mM) and precipitation from 3 M NaOAc, pH = 5.2 in 70% aqueous ethanol. Oligonucleotides were characterized using electrospray ionization mass spectrometry, and purity was assessed by analytical AEX HPLC.

### GalNAc conjugation

Triantennary GalNAc ligand derivatized siRNA was prepared as previously described [19]. In brief, the carboxylic acid precursor dissolved in dimethylformamide (DMF) was activated with *N*-hydroxysuccinimide and diisopropylcarbodiimide at 0°C. This solution was stirred overnight at ambient temperature. Aliquots were added to a 4.4 M solution of amino terminated siRNA in sodium carbonate (pH 9.6):DMSO 2:3 v/v until >85% complete by HPLC. The crude conjugate was precipitated by addition of ethanol and storage in the freezer overnight. *O*-acetyl groups were removed by dissolution in aqueous ammonia at 25°C for 4 h. The product was purified by reverse-phase HPLC.

### Mono-dithiobismaleimidoethane derivatives

Mono-dithiobismaleimidoethane (DTME) derivatives were prepared by treating thiol-modified oligonucleotides [20 OD/mL in 300 mM NaOAc (pH 5.2) containing 25% v/v acetonitrile] with 40 equivalents of DTME (Thermo Fisher, 15.6 mM in acetonitrile) at 25°C with agitation and the reaction monitored by HPLC. On completion, the mono-DTME derivatized oligonucleotides were isolated by size exclusion chromatography with water as eluant or by AEX HPLC and precipitated as before.

DTME-modified oligonucleotides were converted to the corresponding dimers by treatment with a slight molar excess of the relevant thiolated oligonucleotide in 300 mM NaOAc (pH 5.2). This reaction was performed using single-stranded sequences or after prior annealing of the complementary oligonucleotide of one of the reaction partners.

Double-stranded RNAs were generated from RNA single strands by adding a slight molar excess of the required complementary antisense strand(s) to sense strand each in 4 mM sodium phosphate/20 mM NaCl/pH 6.8 buffer at room temperature. Successful duplex formation was confirmed by native size exclusion HPLC using a Superdex 75 column (10 × 300 mm, GE Healthcare). Samples were stored frozen until use.

### Multimer synthesis

Preparation of multimeric siRNAs through stepwise annealing was performed in water and utilized stepwise addition of complementary strands at room temperature with no heating/cooling. Amounts required to combine equimolar amounts of complementary single strands were calculated based on the extinction coefficients for the individual single strands computed by the nearest neighbor method. Annealing was monitored using a Dionex Ultimate 3000 HPLC system equipped with a XBride C18 Oligo BEH (2.5 μm; 2.1 × 50 mm; Waters) column equilibrated to 20°C using hexafluoro-isopropanol (100 mM): triethylamine (16.3 mM) as mobile phase and a gradient of 4.75 to 66% methanol over 30 min. Flow rate was 250 μL/min and diagnostic wavelength was 260 nm.

**Figure:**
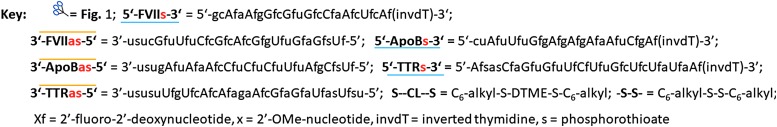


### Animal studies

All procedures using mice were conducted by certified laboratory personnel.

Animal experiments were performed in accordance with the German Animal Protection Law of 2013. All procedures were approved by the local veterinary office (permission no. 55.2DMS-2532-2-38; Regierung von Unterfranken, Wuerzburg, Germany).

C57BL/6N mice were used for all *in vivo* experiments. Animals were obtained from Charles River (Sulzfeld, Germany), and were between 6 and 8 weeks old. LNP formulations were administered intravenously by infusing 200 μL into the lateral tail vein. Subcutaneously administered compounds were injected using a total volume between 100 and 200 μL.

Blood was collected by submandibular vein bleed the day before injection (“prebleed”) and postinjection at indicated times. Serum was isolated by centrifuging blood in serum separation tubes (Greiner Bio-One, Frickenhausen, Germany). Once isolated, serum was frozen until analysis.

Upon killing of the animals, blood was collected by cardiac puncture and serum isolated as described previously. Tissue for mRNA quantification was harvested and immediately snap-frozen in liquid nitrogen. Livers were lysed by sonication and digestion with Proteinase K for 30 min at 65°C in a thermomixer (Thermomixer comfort; Eppendorf, Hamburg, Germany). mRNA levels were quantified using either QuantiGene 1.0 (FVII, ApoB, and GAPDH) or Quantigene 2.0 (TTR) branched DNA (bDNA) Assay Kit (Panomics, Fremont, CA) according to the manufacturer's recommendations. In all cases, mice were anesthetized by CO_2_ inhalation and killed by cervical dislocation.

### Factor VII quantification

Factor VII protein was quantified in serum using the chromogenic enzyme activity assay BIOPHEN FVII (#221304; Hyphen BioMed, MariaEnzersdorf, Austria). Mouse serum was diluted 1:3,000 before analysis. Absorbance of colorimetric development at 405 nm was measured using a Victor 3 multilabel counter (Perkin Elmer, Wiesbaden, Germany).

### Disulfide stability

The single-stranded ApoB C_6_-alkyldisulfide-C_6_alkyl-TTR dimer used in the synthesis of heterotrimer 1 and a newly synthesized equivalent wherein the alkyl chains were linked through phosphothioate linkages were separately added to murine plasma from a 1:1 male:female cohort (Biotrend Chemikalien GmbH, MSEPLEDTA2, Lots: MSE22257 and MSE22256) pretreated with EDTA to prevent coagulation to a final concentration of 50 μM. The resulting solutions were incubated at 37°C. Samples (50 μM) were taken at 0, 1, 2, 4, 8, and 24 h and reactions stopped by addition of DTT-free proteinase K (Sigma) followed by heat inactivation and dilution with water for subsequent IEX HPLC analysis.

### RNA sequences

In all the sequences described in this publication, “X,” “x,” and “Xf” represent a ribonucleotide, 2′-O-methylribonucleotide and 2′-fluoro-2′-deoxyribonucleotide, respectively. “InvdT” represents inverted deoxythymidine residues and “s” represents phosphorothioate linkages. “(SHC_6_)” and “(C_6_SSC_6_)” represent thiohexyl and dihexyldisulfide linkers, respectively. “C_6_NH_2_” and “C_6_NH” are used interchangeably to represent the aminohexyl linker. “(DTME)” represents the cleavable homobifunctional crosslinker DTME.

The specific siRNA sequences used were as follows:

FVII sense: 5′-gcAfaAfgGfcGfuGfcCfaAfcUfcAf(invdT)-3′;

FVII antisense: 3′-usucGfuUfuCfcGfcAfcGfgUfuGfaGfsUf-5′;

TTR sense: 5′-AfsasCfaGfuGfuUfCfUfuGfcUfcUfaUfaAf(invdT)-3′

TTR antisense: 3′-ususuUfgUfcAfcAfagaAfcGfaGfaUfasUfsu-5′

ApoB sense: 5′-cuAfuUfuGfgAfgAfgAfaAfuCfgAf(invdT)-3′

ApoB antisense: 3′-usugAfuAfaAfcCfuCfuCfuUfuAfgCfsUf-5′

## Results

Mixtures of high-molecular-weight multimeric siRNAs of varying lengths were previously reported to increase siRNA potency both *in vitro* and *in vivo* when formulated with highly cationic lipid material to form nanoparticles [20]. The multimers were created by synthesizing both the sense and antisense strands of the siRNA each with a terminal reactive group such as a thiolate, separately crosslinking the sense and antisense strands with a bivalent linking agent such as DTME to form linear homodimers, and then annealing these complementary dimers to form a dynamic mixture of high-molecular-weight species. We first tested whether these high-molecular-weight siRNA mixtures were more effective at silencing target genes than traditional “monomeric” siRNA when formulated into a clinically relevant ionizable LNP, but did not observe any increased silencing relative to a monomeric siRNA control (data not shown). Based on these results, we decided on an alternative approach, making two significant changes. First, we changed the delivery vehicle from an LNP to a covalently conjugated construct. Second, we switched from mixtures of siRNAs, and instead focused on the development of novel synthetic routes to generate multimeric siRNAs of defined length and composition.

**SCHEME 1.**
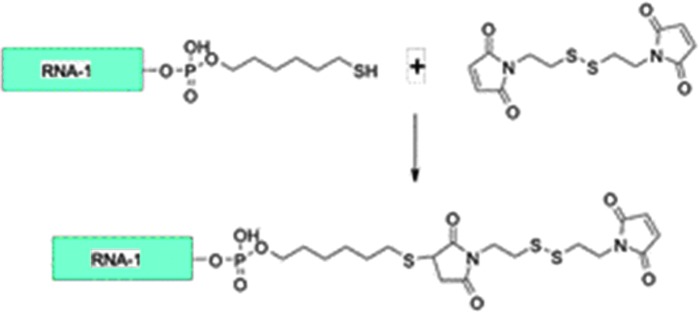
Schematic of general preparation of mono-DTME derivative of an oligonucleotide. DTME, dithiobismaleimidoethane.

During this study we made an exciting observation: that it was possible to prepare monosubstituted DTME derivatives of oligonucleotides in high yield by treatment of a terminally thiolated oligo with a large excess of DTME (Scheme 1).

These mono-DTME derivatives could then be subsequently reacted with other terminally thiolated siRNAs in aqueous solution, enabling the facile production of asymmetric homo- and heteromultimers in high yield. Of note, the multimers could be synthesized in any orientation, including 3′-3′ and 5′-5′ linked oligos, which have been difficult to synthesize using other means. Furthermore, these multimers would be converted to the corresponding monomeric units of siRNA under reducing intracellular conditions because of the presence of the internal disulfide group in the DTME moiety. As a result, these mono-DTME derivatives became key intermediates in our subsequent study.

**FIG. 1.**
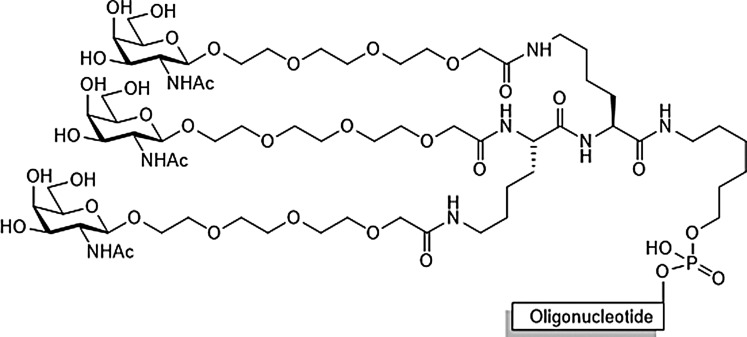
Structure of triantennary GalNAc ligand. FalNAc, *N*-acetylgalactosamine.

Our next aim was to determine how the positioning of the linker and ligand affected the gene silencing of a multimeric siRNA. We performed these studies using as a model system (i) a version [19] (Fig. 1) of a triantennary GalNAc ligand because of its well-established affinity for ASGPR and the ability to deliver siRNA into the cytoplasm of hepatocytes *in vitro* and *in vivo* and (ii) an siRNA previously used [21] to achieve knockdown of FVII, a well-known protein marker with gene expression limited specifically to hepatocytes. To evaluate how the positioning of the linker affected gene silencing, we prepared three isomeric GalNAc-FVII homodimers, that is, defined constructs with two siRNAs containing the same target sequence.

Of note, each siRNA contained chemically modified nucleosides in a pattern previously shown [22] to promote on-target gene silencing, reduce immune stimulation, and increase RNA stability (Materials and Methods).

**FIG. 2.**
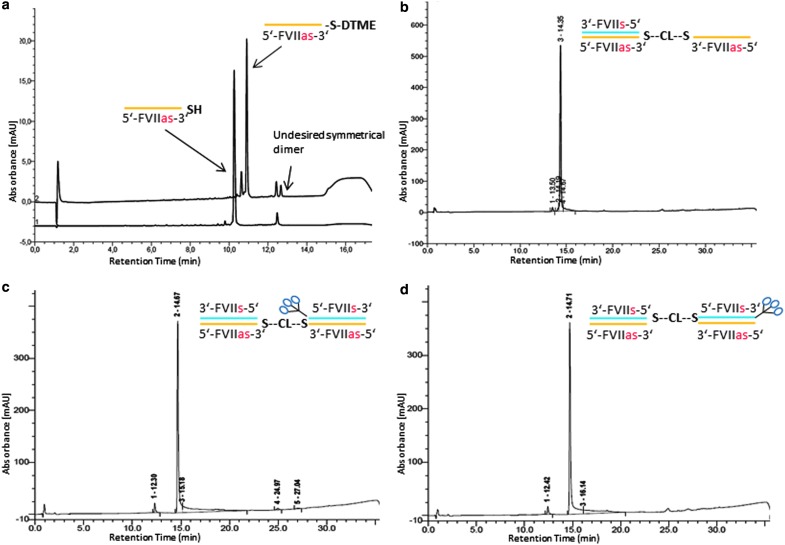
HPLC chromatograms of intermediates in synthesis of homodimers 1 and 2: **(a)** formation of FVII-antisense mono-DTME derivative, **(b)** “half-dimer duplex,” **(c)** isolated homodimer 1, and **(d)** homodimer 2. DTME, dithiobismaleimidoethane; HPLC, high-performance liquid chromatography.

**SCHEME 2.**
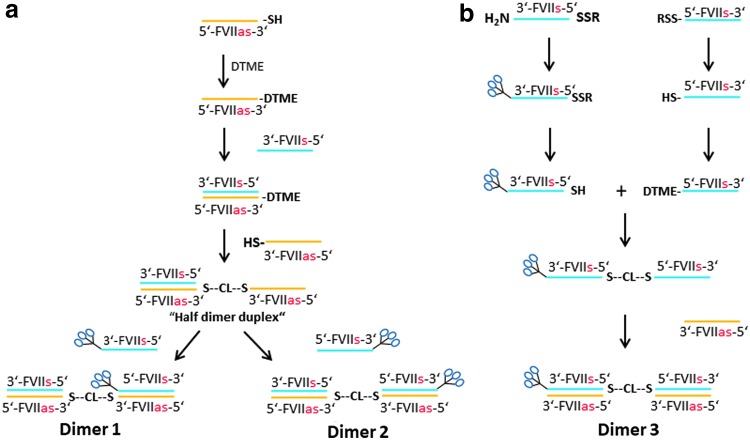
Preparation of isomeric GalNAc-FVII homodimers **(a)** 1, 2, and **(b)** 3 by mono-DTME derivatives.

We synthesized the first two GalNAc FVII homodimers using a mono-DTME derivative of the antisense strand of FVII (Scheme 2a). First, we converted the starting thiol-terminated antisense FVII sequence into the mono-DTME derivative using a 40-fold excess of DTME. We found that under these conditions, the formation of the undesired symmetrical homodimer was minimized (Fig. 2a). Addition of the corresponding sense sequence formed a double-stranded monomeric siRNA with a DTME linker on the 3′ end of the antisense strand that was then reacted with 1 molar equivalent (mol. equiv.) of the thiol-terminated antisense strand to yield the “half dimer duplex” that was isolated using HPLC in 59% yield in high purity (Fig. 2b). On treatment with one equivalent of complementary sense FVII RNAs with GalNAc conjugates on the 5′ or 3′ end in two separate reactions the half dimer duplex was completely converted into the isomeric GalNAc FVII homodimers 1 and 2 as monitored by HPLC analysis (Fig. 2c, d).

**FIG. 3.**
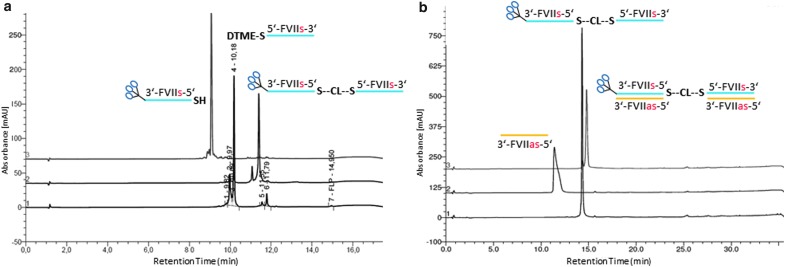
HPLC chromatograms of intermediates in synthesis of homodimer 3: **(a)** reaction of thiolated FVII with FVII-mono-DTME derivative, **(b)** subsequent annealing with two FVII strands to form homodimer 3.

GalNAc-FVII homodimer 3 was prepared from a 5′-thiolated FVII sense strand by the corresponding mono-DTME derivative (Scheme 2b) and subsequent addition of 1 mol. equiv. of a 3′-GalNAc 5′-thiolated FVII strand to yield the 3′-GalNAc FVII single-stranded homodimer in 5′-5′ orientation in 64% yield (Fig. 3a). This material was completely converted by addition of 2 mol. equiv. of the FVII antisense strand into the desired double-stranded GalNAc FVII homodimer 3 as monitored by HPLC analysis (Fig. 3b).

Based on these results, we hypothesized that it might be possible to prepare larger heteromultimers to facilitate the knockdown of multiple mRNA targets. Once again, we used the triantennary GalNAc ligand (Fig. 1). In addition to FVII siRNA we added siRNAs targeting mRNAs encoding for transthyretin (TTR) and ApoB. A siRNA sequence targeting TTR was previously reported [23]; however, a murine ApoB siRNA sequence with sufficient chemical modifications to be deliverable by GalNAc had not been reported. To this end, we used bioinformatic analysis to identify 12 sequences, each targeting ApoB mRNA. We then transfected the sequences into NMuLi cells, which express ApoB, and screened for ApoB knockdown (data not shown). From the resulting data, the sequences GGAAUCuuAuAuuuGAUCcAsA and uuGGAUcAAAuAuAAGAuUCcscsU were chosen as ApoB sense and antisense strands, respectively (Key Figure in Materials and Methods section).

**FIG. 4.**
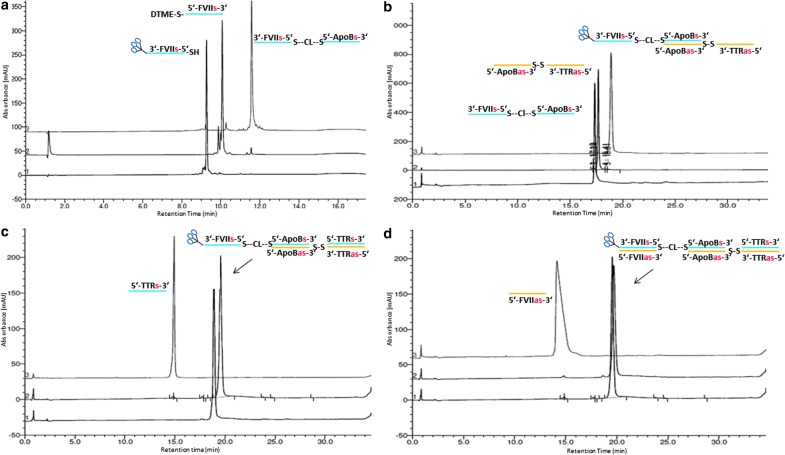
HPLC chromatograms of intermediates in synthesis of heterotrimer 1: **(a)** formation of FVIIs-ApoB heterodimer by mono-DTME derivative, **(b)** asymmetric annealing of single-stranded dimer from **(a)** with second single-stranded dimer ex-synthesizer, **(c)** annealing of TTR sense strand and **(d)** annealing of FVII sense strand to form heterotrimer 1. TTR, transthyretin.

**SCHEME 3.**
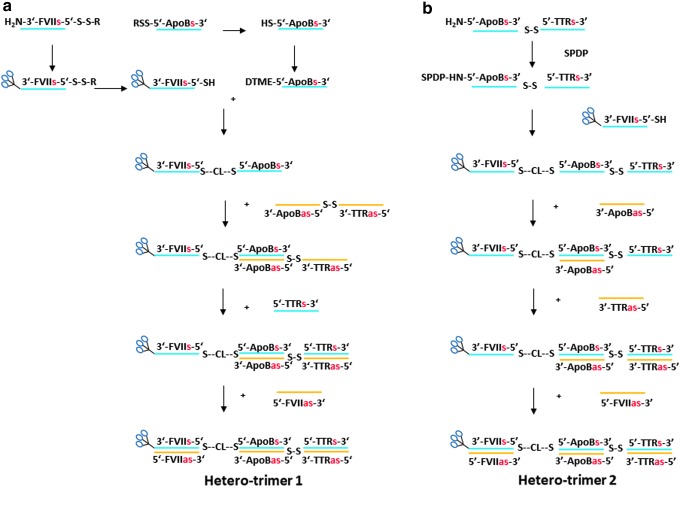
Preparation of isomeric GalNAc-FVII-ApoB-TTR-heterotrimers **(a)** 1 and **(b)** 2 by mono-DTME derivative and asymmetric annealing of single-stranded dimers.

We then prepared heterotrimer 1 containing siRNAs targeting *FVII*, *ApoB*, and *TTR* with linkers on alternating strands through an asymmetric annealing approach to take advantage of the specific binding ability of complementary sense and antisense strands (Scheme 3a). This material was prepared by a 3′-GalNAc FVII-ApoB single-sense-stranded heterodimer, prepared through a mono-DTME derivative that was then reacted with thiolated GalNAc siRNA in similar fashion to the homodimers prepared previously and isolated in 73% overall yield and high purity (Fig. 4a). In this case, however, the 3′-GalNAc FVII-ApoB heterodimer was then asymmetrically annealed under neutral aqueous conditions at room temperature with 1 mol. equiv. of a single-antisense-stranded ApoB-TTR heterodimer prepared on the synthesizer. Subsequent equimolar annealing of one equivalent each of TTR sense and FVII antisense strands afforded heterotrimer 1 with disulfide linkages on alternating chains. All annealing steps proceeded to completion as monitored by HPLC analysis to provide heterotrimer 1 in high purity (Fig. 4b–d).

After completing the synthesis of this initial heterotrimer, we then synthesized the isomeric GalNAc heterotrimer 2. In this case, the heterotrimer contained two disulfide linkages on the sense strands (Scheme 3b). This molecule was synthesized by treating an amino-terminated ApoB-TTR heterodimer with succinimidyl 3-(2-pyridyldithio)propionate and reacting the resulting derivative with thiolated FVII sense strand X18793 to yield the single-stranded FVII-ApoB-TTR heterotrimer in 41% yield with respect to the starting ApoB-TTR heterodimer. This compound, in turn, was sequentially annealed using 1 mol. equiv. of ApoB, TTR, and FVII antisense strands to yield heterotrimer 2. Again, each annealing step proceeded to completion as monitored by HPLC analysis (data not shown).

**FIG. 5.**
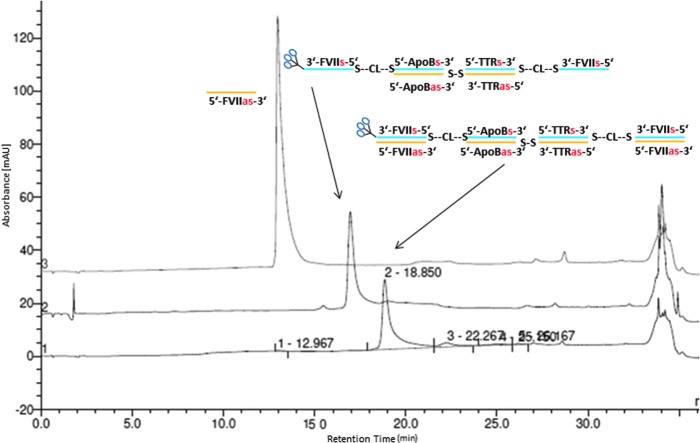
Overlay of HPLC chromatograms of intermediates in synthesis of heterotetramer showing addition of 2 mol. equiv. of FVII antisense strands to the product of two asymmetric annealing reactions.

**SCHEME 4.**
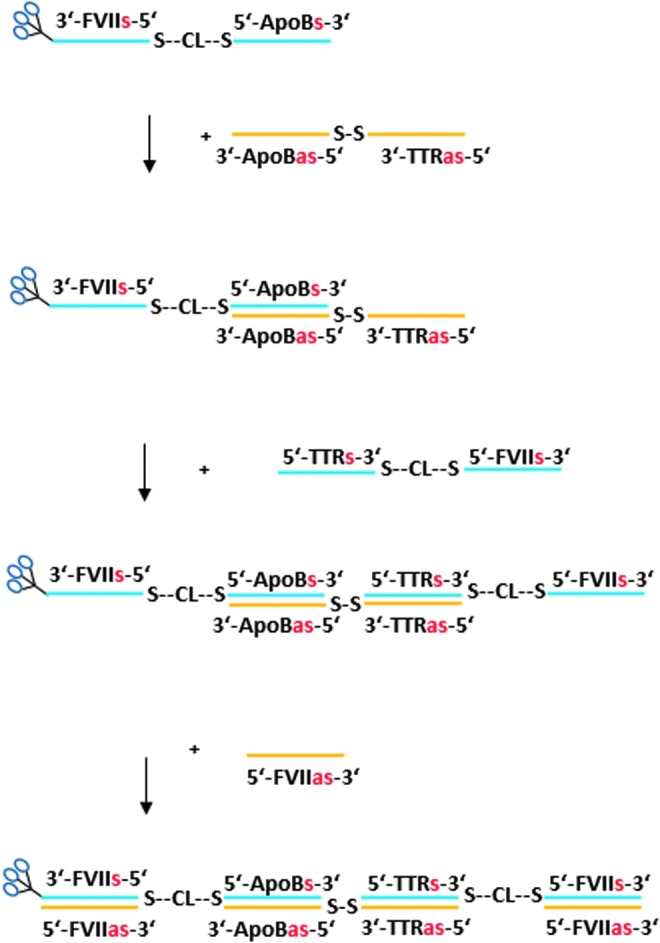
Preparation of GalNAc-FVII-ApoB-TTR-heterotetramer by mono-DTME derivative and two asymmetric annealings.

To demonstrate that even larger multimers may be efficiently prepared by the asymmetric annealing technique, a heterotetramer containing two units of FVII and one each of ApoB and TTR siRNA was prepared (Scheme 4) through two sequential asymmetric annealings, wherein a single-stranded GalNAc FVII-ApoB heterodimer was annealed first with an ApoB-TTR heterodimer prepared on the synthesizer, and the product annealed with a TTR-FVII heterodimer that was prepared through a mono-DTME derivative. Subsequent addition of 2 mol. equiv. of monomeric FVII antisense strand afforded a GalNAc heterotetramer containing FVII:ApoB:TTR siRNAs in the molar ratio 2:1:1 (Fig. 5).

**FIG. 6.**
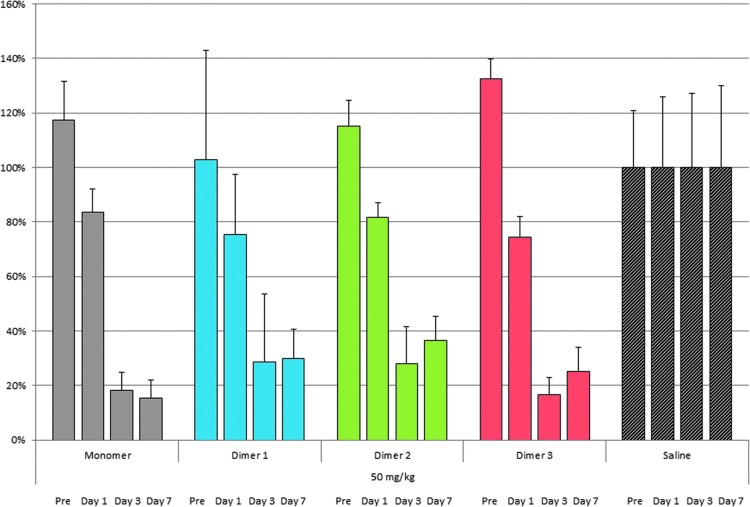
Knockdown of FVII activity by homodimers 1, 2, and 3 and monomeric control versus saline at days 1, 3, and 7 at 50 mg/kg.

**FIG. 7.**
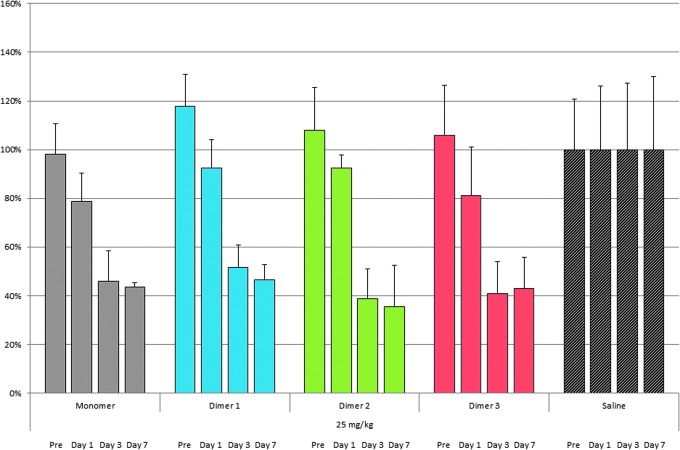
Knockdown of FVII activity by homodimers 1, 2 and 3 and monomeric control versus saline at days 1, 3, and 7 at 25 mg/kg.

We then tested the ability of the three isomeric homodimers to functionally deliver siRNA to hepatocytes *in vivo.* We subcutaneously injected female C57BL/6 mice with 25 or 50 mg/kg doses of each of the three GalNAc homodimers, using five mice per treatment group. We included two controls: mice injected with PBS, which served as the negative control, and mice injected with monomeric GalNAc-FVII, which served as the positive control. FVII serum activity was measured 1 day before injection, and 1, 3, and 7 days after injection. The FVII serum data obtained strongly suggested that adding a second siRNA did not inhibit GalNAc-mediated delivery. Specifically, the FVII homodimers reduced FVII serum protein levels approximately as potently as the monomeric siRNA-positive controls (Figs. 6 and 7). Relative to saline-treated control mice, these reductions in FVII serum levels were highly significant (0.011 > *P* > 0.001). We found no significant difference in FVII silencing between the monomeric control and homodimers 1, 2, and 3 at 25 mg/kg doses, nor between the control and dimers 1 and 2 at 50 mg/kg doses. We did observe that the monomeric control outperformed homodimer 3 construct at 50 mg/kg.

**FIG. 8.**
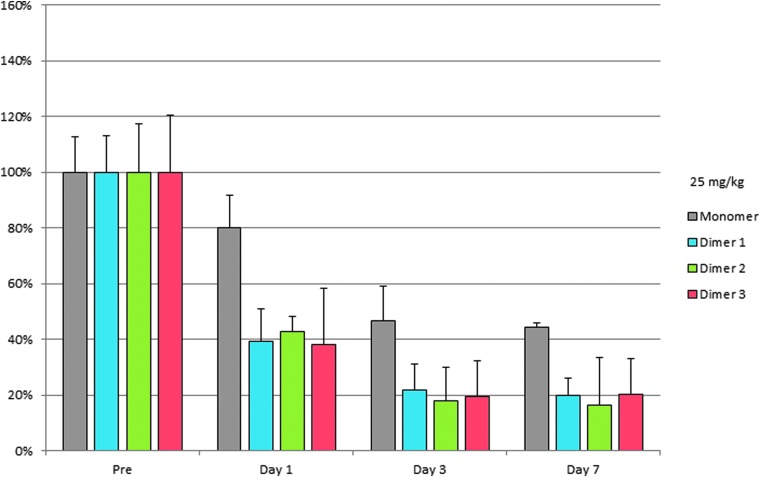
Knockdown of FVII activity by FVII siRNA in homodimers 1, 2, and 3 and monomeric control versus saline at days 1, 3, and 7 at 25 mg/kg per mole of GalNAc ligand. siRNA, small interfering RNA.

Furthermore, the silencing per mole of GalNAc ligand in the homodimer groups was approximately twice that of the monomeric group at 25 mg/kg (Fig. 8) demonstrating that at the doses used a single GalNAc unit can efficiently deliver two siRNA units in active form *in vivo*. Of note, the results were consistent, independent of the linker and ligand positions, suggesting that these did not dramatically alter siRNA delivery.

We also quantified aspartate aminotransferase (AST) and alanine aminotransferase (ALT) levels in the test mice 1 and 3 days after administration of homodimers 1, 2, and 3 and the monomeric control. We found no statistically significant increases in either liver enzyme in mice treated with any of the siRNA constructs, relative to mice treated with PBS (data not shown).

We then determined whether GalNAc-mediated delivery could silence multiple genes at once. We administered the two heterotrimeric GalNAc-conjugated siRNAs each targeting *FVII*, *ApoB*, and *TTR* to four female 7-week-old C57BL/6 mice at dosages of 50 mg/kg (ie, equivalent to 17 mg/kg of each siRNA). We included saline as a negative control and a pool of three monomeric GalNAc-conjugated siRNAs targeting *FVII*, *ApoB*, and *TTR*, respectively, each at 17 mg/kg as positive control. Blood was collected at −1, 1, 3, and 7 days and serum levels of FVII, ApoB, and TTR proteins were measured. mRNA levels in liver lysates were measured at day 7 postinjection.

**FIG. 9.**
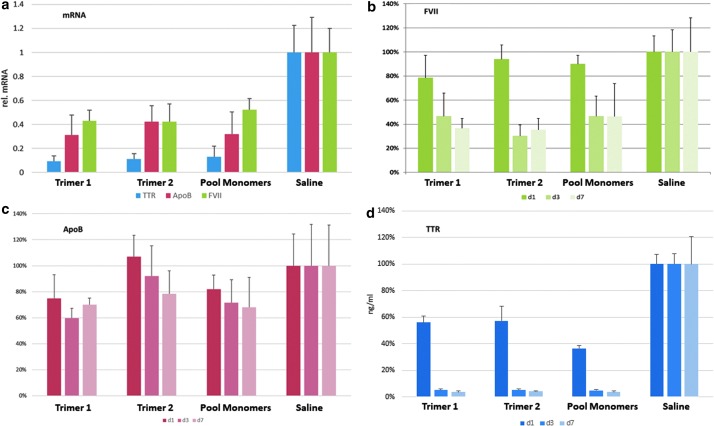
Knockdown of **(b)** FVII, **(d)** TTR, and **(c)** ApoB proteins and **(a)** mRNAs by heterotrimers 1 and 2 (50 mg/kg) and pool of monomeric controls (17 mg/kg each) versus saline at days 1, 3, and 7.

Once again, knockdown per unit of each siRNA at each time point was essentially the same in each of the multimers, relative to the corresponding pooled GalNAc monomers (Fig. 9a–d). Reduction of FVII concentrations on days 3 and 7 were highly significant (*P* < 0.004, two-tailed Student's *t*-test). All the delivery systems led to highly significant reductions in serum TTR at 1, 3, and 7 days after administration with no statistical difference between the individual GalNAc controls and heterotrimers 1 and 2 at any time point, except for the fact that the individual control outperformed trimer 2 on day 1. We also measured ApoB silencing; when compared with both FVII and TTR silencing, serum ApoB silencing was less robust for all tested groups at all tested timepoints.

All these knockdowns were achieved by the trimers with one-third the proportion of GalNAc ligand relative to the monomer controls, again suggesting that at the doses employed a single GalNAc unit can efficiently deliver active multimeric siRNA cargos *in vivo* and further can facilitate the simultaneous targeting of multiple transcripts. We consider this may have significant utility in diseases where knockdowns of multiple pathways are required for optimum effect and/or when differing types of oligonucleotide agent (eg, siRNA and miRNA) are required.

**FIG. 10.**
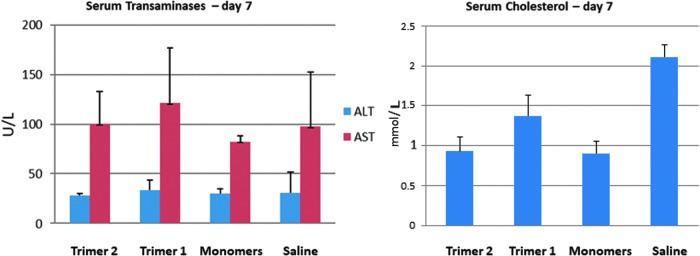
Transaminase and cholesterol levels in serum after administration of heterotrimers 1 and 2 and pool of monomeric controls versus saline at day 7.

We also analyzed ALT and AST levels in serum samples taken 7 days after administration of the two heterotrimers, the pool of GalNAc monomers and the saline control. We found no significant increases in levels of ALT or AST (Fig. 10) in any of the samples tested, which suggests the heterotrimers did not lead to hepatic dysfunction at the tested doses. Reduction in cholesterol levels was observed in all three test samples (Fig. 10).

**FIG. 11.**
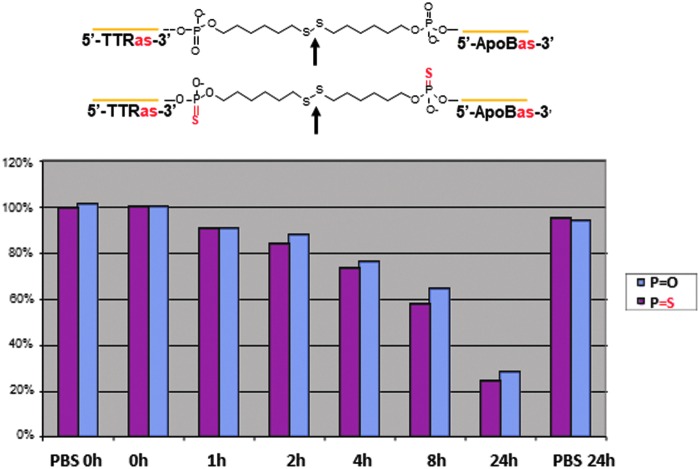
Stability of linkers used in preparation of homodimers and heterotrimers. Determination of concentration over time in murine plasma of heterodimer used in synthesis of heterotrimer 1 (*blue bars*) and a phosphothioate analogue (*maroon bars*). Points of initial cleavage in the structures are indicated by the bold *arrows*.

Finally, we tested the stability in murine plasma of the C_6_SSC_6_ linkers used in preparation of both heterotrimers. The ApoBas-TTRas single-stranded dimer used in the preparation of Trimer 1, and an analogue wherein the phosphodiester groups at both ends of the linker were replaced with phosphothioate groups were separately treated with murine plasma at 37°C and samples taken hourly. HPLC analysis showed that both constructs were equally stable, both degrading at essentially the same rate with a half-life of ∼10 h (Fig. 11). These data suggest that initial cleavage was occurring at the central disulfide bond rather than at the phosphate ester termini, a finding supported by mass spec analysis of the initial breakdown products (data not shown).

## Conclusions

Taken together the data indicate that ligand directed multimeric oligonucleotide drugs may be easily prepared in high yield and purity under mild aqueous conditions, and that for multimers up to at least trimers in size, multiple therapeutic units can be delivered by GalNAc ligand with no loss of activity of the individual units and with little or no detectable toxicity. We believe the potential for delivery of multiple units of siRNA may be of critical importance for effectively targeting other cell/receptor types where ligand affinity and/or receptor copy number and internalization rate are not optimum, and/or diseases where knockdowns of multiple pathways are required for optimum effect. Work continues in determining the properties and associated advantages of still larger ligand-directed multimers.
